# Supplementary figures and images for: Microglial Morphological Complexity in the Piriform Cortex Is Associated with Olfactory Aversion Following Chronic Stress
Source: eNeuro. 2026 May 5;13(5):ENEURO.0330-25.2026. doi: 10.1523/ENEURO.0330-25.2026 (PMC13159971; doi:10.1523/ENEURO.0330-25.2026)

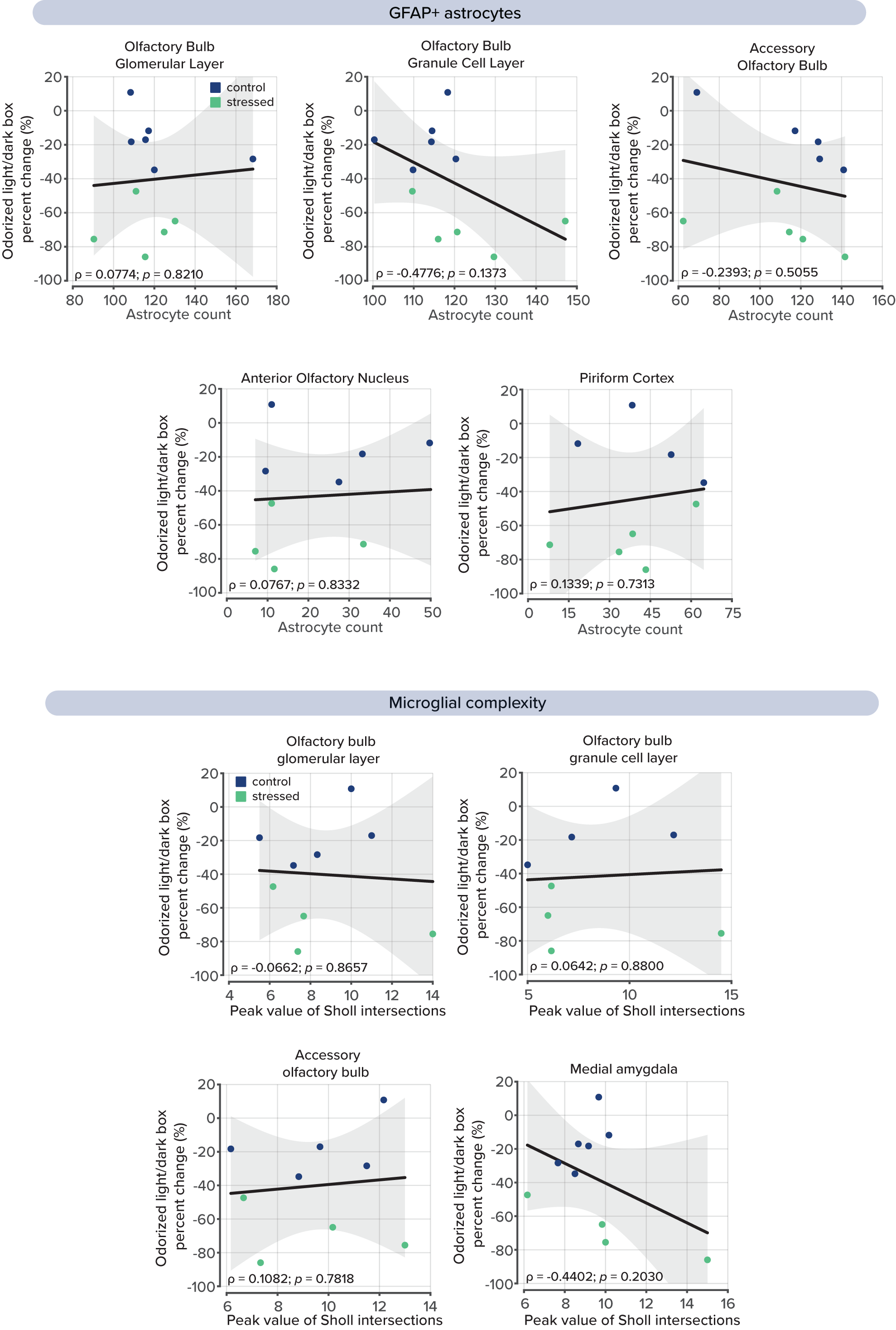

Supplement: Figure 6-1 — Correlation analysis between labeled astrocytes and odorant aversion in the olfactory bulb glomerular layer, olfactory bulb granule cell layer, accessory olfactory bulb, anterior olfactory nucleus, and anterior piriform cortex. Download Figure 6-1, TIF file. [file eneuro-13-ENEURO.0330-25.2026-s002.tif]
