# Supplementary material for: Microglial Morphological Complexity in the Piriform Cortex Is Associated with Olfactory Aversion Following Chronic Stress
Source: eNeuro. 2026 May 5;13(5):ENEURO.0330-25.2026. doi: 10.1523/ENEURO.0330-25.2026 (PMC13159971; doi:10.1523/ENEURO.0330-25.2026)
Supplement: Figure 6-2 — Pearson correlations between odor avoidance behavior and astrocyte counts per animal, pooled across groups. Download Figure 6-2, DOCX file. [file eneuro-13-ENEURO.0330-25.2026-s003.docx]

| Region | ρ | t | *n* | *p*-value | Sig. |
| --- | --- | --- | --- | --- | --- |
| Olfactory bulb glomerular layer | +0.0774 | +0.2329 | 11 | 0.8210 | ns |
| Olfactory bulb granule cell layer | −0.4776 | −1.6310 | 11 | 0.1373 | ns |
| Accessory olfactory bulb | −0.2302 | −0.6690 | 10 | 0.5223 | ns |
| Anterior olfactory nucleus | +0.0767 | +0.2176 | 10 | 0.8332 | ns |
| Anterior piriform cortex | +0.1339 | +0.3574 | 9 | 0.7313 | ns |
| Medial amygdala | −0.7050 | −2.8118 | 10 | 0.0228 | * |
