# Supplementary material for: Microglial Morphological Complexity in the Piriform Cortex Is Associated with Olfactory Aversion Following Chronic Stress
Source: eNeuro. 2026 May 5;13(5):ENEURO.0330-25.2026. doi: 10.1523/ENEURO.0330-25.2026 (PMC13159971; doi:10.1523/ENEURO.0330-25.2026)
Supplement: Figure 6-3 — Pearson correlations between odor avoidance behavior and peak Sholl intersections per animal, pooled across groups. Download Figure 6-3, DOCX file. [file eneuro-13-ENEURO.0330-25.2026-s004.docx]

| Region | ρ | t | *n* | *p*-value | Sig. |
| --- | --- | --- | --- | --- | --- |
| Olfactory bulb glomerular layer | −0.0662 | −0.1755 | 9 | 0.8657 | ns |
| Olfactory bulb granule cell layer | +0.0642 | +0.1575 | 8 | 0.8800 | ns |
| Accessory olfactory bulb | +0.1082 | +0.2878 | 9 | 0.7818 | ns |
| Anterior olfactory nucleus | −0.6759 | −2.5942 | 10 | 0.0319 | * |
| Anterior piriform cortex | −0.8279 | −4.1751 | 10 | 0.0031 | ** (FDR) |
| Medial amygdala | −0.4402 | −1.3865 | 10 | 0.2030 | ns |
